# Thyroid Hormone Reverses Aging-Induced Myocardial Fatty Acid Oxidation Defects and Improves the Response to Acutely Increased Afterload

**DOI:** 10.1371/journal.pone.0065532

**Published:** 2013-06-07

**Authors:** Dolena Ledee, Michael A. Portman, Masaki Kajimoto, Nancy Isern, Aaron K. Olson

**Affiliations:** 1 Seattle Children's Research Institute, Seattle, Washington, United States of America; 2 Division of Cardiology, Department of Pediatrics, University of Washington, Seattle, Washington, United States of America; 3 Environmental Molecular Sciences Laboratory (EMSL), Pacific Northwest National Laboratory, Richland, Washington, United States of America; Max Delbrueck Center for Molecular Medicine, Germany

## Abstract

**Background:**

Subclinical hypothyroidism occurs during aging in humans and mice and may contribute to the development of heart failure. Aging also impairs myocardial fatty acid oxidation, causing increased reliance on flux through pyruvate dehydrogenase (PDH) to maintain function. We hypothesize that the metabolic changes in aged hearts make them less tolerant to acutely increased work and that thyroid hormone supplementation reverses these defects.

**Methods:**

Studies were performed on young (Young, 4–6 months) and aged (Old, 22–24 months) C57/BL6 mice at standard (50 mmHg) and high afterload (80 mmHg). Another aged group received thyroid hormone for 3 weeks (Old-TH, high afterload only). Function was measured in isolated working hearts along with substrate fractional contributions (Fc) to the citric acid cycle (CAC) using perfusate with ^13^C labeled lactate, pyruvate, glucose and unlabeled palmitate and insulin.

**Results:**

Old mice maintained cardiac function under standard workload conditions, despite a marked decrease in unlabeled (presumably palmitate) Fc and relatively similar individual carbohydrate contributions. However, old mice exhibited reduced palmitate oxidation with diastolic dysfunction exemplified by lower -dP/dT. Thyroid hormone abrogated the functional and substrate flux abnormalities in aged mice.

**Conclusion:**

The aged heart shows diminished ability to increase cardiac work due to substrate limitations, primarily impaired fatty acid oxidation. The heart accommodates slightly by increasing efficiency through oxidation of carbohydrate substrates. Thyroid hormone supplementation in aged mice significantly improves cardiac function potentially through restoration of fatty acid oxidation.

## Introduction

Subclinical hypothyroidism is characterized by increased serum thyrotropin (thyroid stimulating hormone, TSH) concentration in the presence of normal values of free thyroxin (T4) and biologically active free triiodothyronine (T3). This condition is especially common in the elderly with a prevalence of up to 10% [1]. While not present in every study, multiple investigations have demonstrated an association between subclinical hypothyroidism and an increased risk of congestive heart failure [2], [3], [4]. Thyroxin replacement improved measures of cardiac function in a small study [5]; however no large randomized controlled trials have been completed. The mechanisms for this therapeutic effect still require elucidation. Thyroid hormone regulates excitation-contraction and transport proteins such as sarcoplasmic reticulum calcium ATPase (SERCA2), α-myosin heavy chain, β1 adrenergic receptors, sodium/potassium ATPase, voltage-gated potassium channels, malic enzyme and atrial and brain natriuretic factor [6], [7]. Since these proteins form the primary architecture of the cardiomyocyte and determine contractile strength, modulation of these proteins by thyroid hormone is a biologically plausible mechanism for improvements in cardiac function noted in the elderly after thyroid hormone supplementation. However, contractile function also depends upon energy supply. Modifications in substrate oxidation within the mitochondria influence cardiomyocyte environment and structure. Metabolic abnormalities as etiologies for cardiomyopathy are well recognized [8], [9], but exploration has only started with any detail in aging models.

Several experimental studies have demonstrated alteration in myocardial oxidative metabolism from aging [10], [11]. McMillin et al. showed that the senescent rat heart lacked the ability to suppress glucose oxidation when supplied with oleate; whereas Koonen et al. showed an increase in the fractional contribution (Fc) of palmitate to the citric acid cycle in 50–52 week old mice compared to younger mice although actual palmitate oxidation was decreased [12], [13]. These studies did not employ the robust substrate supply conditions present in vivo, therefore their applicability is unclear. Accordingly, we previously investigated oxidative metabolism in aged mouse hearts perfused with glucose, lactate, ketones and free fatty acids [14]. Ketone and free fatty acid oxidation decreased with aging while glucose oxidation reciprocally increased. This study demonstrated that the aged hearts were able to maintain function by increasing carbohydrate utilization. However, anaplerosis was impaired in aged hearts, potentially limiting the response to acute stress through depletion of citric acid cycle intermediates. Thyroid hormone levels were reduced in the aged mice, suggesting that myocardial metabolic alterations may play a pathologic role in subclinical hypothyroidism.

Our primary hypothesis is that disruptions in thyroid hormone homeostasis affect the metabolic phenotype in the aged heart and contribute to the cardiomyopathy of aging. Specifically, we tested whether thyroid hormone (TH) supplementation alters substrate utilization for the citric acid cycle in the aged heart and makes it more tolerant to acutely increased afterload. Our prior study focused primarily on shifts in fatty acid oxidation and anaplerosis with limited data reflecting carbohydrate oxidation [14]. Accordingly, we focused on carbohydrate oxidative metabolism for the current study.

## Materials and Methods

### Ethics Statement

This investigation conforms to the Guide for the Care and Use of Laboratory Animals published by the National Institute of Health (NIH Pub. No. 85–23, revised 1996) and were reviewed and approved by the Office of Animal Care at Seattle Children’s Research Institute.

### Animals

This study used C57/Bl6 mice in two age groups purchased from Charles Rivers Laboratories International, Inc (Wilmington, MA). The Young and Old groups consisted of 4–6 and 22–24 month old mice, respectively.

### Thyroid Hormone Administration

Circulating thyroid hormone levels modestly decline with age in mice [14]. To test whether this affects myocardial substrate utilization and function, a cohort of old mice received Thyroid Powder-Grade III (product number T6384, Sigma-Aldrich) for 3 weeks prior to studies (group Old-TH). 0.378 mg daily of Thyroid Powder was administered with the standard chow diet. This dose was based upon a work by Pantos et al. [15]. This thyroid powder has a 10-fold higher ratio of T3 to T4 than present in vivo.

### Thyroid Hormone and Free Fatty Acid Levels

Thyroid hormone levels were determined at the Hormone Assay and Analytical Services Core at Vanderbilt University Medical Center. T3 and T4 levels were determined using a 5-day double antibody assay of plasma. Serum fatty acids were determined using the Free Fatty Acid Assay Kit (Cayman Chemical Company, Ann Arbor, MI).

### Echocardiogram

Serial echocardiograms were performed on a cohort of Old mice before and after 3 weeks of Thyroid Powder. Mice were initially sedated with 3% isoflurane in 21% O_2_ at a flow of 1 LPM and placed in a supine position at which time the isoflurane is reduced to 1% administered via a small nose cone. ECG leads were placed for simultaneous ECG monitoring during image acquisition. Echocardiographic images were performed with a Vevo 2100 machine using a MS400 transducer (VisualSonics, Inc, Toronto, Canada). M-Mode measurements at the midpapillary level of the left ventricle (LV) were performed at end-diastole (LVEDD) and end-systole (LVESD) to determine LV function via the fractional shortening [(LVEDD-LVESD)/LVEDD * 100] in a parasternal short axis mode. Measurements were taken from a parasternal short axis view or a 4 chamber view (mitral valve flow velocities).

### Isolated Working Heart Preparation

Experiments were performed as previously described with some minor changes noted below [16]. Briefly, mice were heparinized (5000 U/kg ip) and anesthetized with a mixture of ketamine (90 mg/kg) and xylazine (10 mg/kg). After adequate sedation, the aorta was rapidly cannulated, excised and placed on the perfusion system. The hearts were initially perfused in a Langendorff manner (70 mmHg perfusion pressure) with physiological salt solution (PSS), pH 7.4, containing (in mmol/l) 118.0 NaCl,25.0 NaHCO_3_, 4.7 KCl, 1.23 MgSO_4_, 1.2 NaH_2_PO_4_, 5.5 D-glucose, and 1.2 CaCl_2_. During retrograde perfusion, the left atrium was cannulated and connected to the preload reservoir set at 15 mm Hg. These hearts were initially stabilized at 50 mmHg afterload for 10 minutes before acutely increasing afterload to 80 mmHg. For comparison, a limited number of mice were perfused with standard loading conditions consisting of a 12 mmHg preload and a 50 mmHg afterload (termed standard afterload because this is the usual afterload for murine working heart experiments in our lab). An SPR-PV-Catheter (SPR-869 or -839 Millar Pressure-Volume Systems, Millar Instruments, Inc, Houston, TX) was inserted into the left ventricle through the apex for continuous measurement of left ventricular pressure (LVP). The hearts were then switched to the antegrade work-performing mode with perfusion into the left atrium of semi-recirculating PSS with the ^13^C-labeled metabolic substrates discussed below. Only aortic outflow that bypasses the coronary arteries is recirculated. Left atrial inflow was measured with a flow probe (T403; Transonic Systems, Ithaca, NY) and aortic (not including coronary flow) flow was measured via 30 second timed collections. Coronary flow was calculated as the difference between left atrial inflow and aortic flow although this measurement is affected by perfusate leak from the left atrium. Every 10 minutes, left atrial influent and coronary effluent was collected for determination of PO_2_, PCO_2_, and pH with an ABL800 blood gas analyzer (Radiometer, Copenhagen, Denmark). Continuously recorded parameters are left ventricular (LV) pressure (mmHg), HR (beats/min), and rate of LV contraction and relaxation (±dP/dt, mmHg/s). Developed pressure (DP) was calculated as the maximum LV pressure subtracted by LV end diastolic pressure. Cardiac work was calculated as the cardiac output times the developed pressure. Myocardial oxygen consumption (MVO_2_) was calculated as MVO_2_ = CF×[(Pa_O2_−Pv_O2_)×(*c*/760)]×heart weight, where CF is coronary flow (ml·min^−1^g wet weight^−1^), (Pa_O2_−Pv_O2_) is the difference in the partial pressure of oxygen (P_O2_, mmHg) between perfusate and coronary effluent, and *c* is the Bunsen solubility coefficient of O2 in perfusate at 37°C (22.7 µl O_2_·atm^−1^·ml^−1^). Cardiac efficiency was defined as cardiac work/MVO_2_.

### Experimental Protocol for Working Hearts

As noted, we wanted to detail carbohydrate oxidation. Accordingly, we perfused the hearts with PSS solution with insulin and ^13^C-labeled substrates in addition to unlabeled sodium palmitate (0.3 mmol/l): [1-^13^C] glucose (glucose, 5.5 mmol/l), [U-^13^C]sodium lactate (lactate, 1.2 mmol/l) and [2-^13^C] sodium pyruvate (pyruvate, 0.2 mmol/l). The rationale for this perfusion strategy was previously employed by Lloyd et al [17]. The palmitate was bound to 0.75% (wt/vol) delipidated bovine serum albumin reconstituted with deionized water. We performed working heart experiments in Young and Old mice. To test whether TH reverses metabolic and functional changes from aging, another group of Old mice received TH supplementation for 3 weeks (Old-TH) prior to working heart experiments.

### ^13^C Magnetic Resonance Spectroscopy (MRS) and Isotopomer Analyses to Determine Substrate Utilization for the Citric Acid Cycle

Myocardial tissue was extracted as previously described [18]. Substrate metabolism was established by using ^13^C-labeled substrates in combination with NMR spectroscopy. Glutamate isotopomer analyses provide Fc of acetyl-CoA to the citric acid cycle from up to three differentially labeled substrates as well as the unlabeled components.

Lyophilized heart extracts were dissolved in 99.8% D2O for decoupled ^13^C NMR spectral acquisition. NMR free-induction decays (FIDs) were acquired on a Varian Direct Drive (VNMRS) 600 MHz spectrometer (Varian Inc., Palo Alto, CA) equipped with a Dell Precision 390 Linux workstation running VNMRJ 2.2C. The spectrometer system was outfitted with a Varian triple resonance salt-tolerant cold probe with a cold carbon preamplifier. A Varian standard one dimensional carbon direct observe sequence with proton decoupling was used to collect data on each sample. Final spectra were accumulations of 4800 individual FIDs. Each FID was induced using a nonselective, 45-degree excitation pulse (7.05 us @ 58 dB), with an acquisition time of 1.3 seconds, a recycle delay of 3 seconds, and a spectral width of 224.1 ppm.

FIDs were baseline corrected, zero-filled, and Fourier transformed. All of the labeled carbon resonances (C1–C5) of glutamate were integrated with the Lorentzian peak fitting subroutine in the acquisition program (NUTS, Acorn NMR, Livermore, CA). The individual integral values were used as starting parameters for the citric acid cycle analysis fitting algorithm tcaCALC, kindly provided by Drs. Mallow and Jeffrey [19]. This algorithm provided the Fc for each substrate in the acetyl-CoA pool entering citric acid cycle. The absolute flux for the citric acid cycle and oxidative flux for individual substrates were calculated from MVO_2_ and the stoichiometric relationships between oxygen consumption and citrate formation from the various substrates as described by Jeffrey et al [20]. The calculated value accounts for changes in oxidative rates, as well as the anaplerotic contribution to the citric acid cycle. Briefly, MVO_2_/citric acid cycle_flux_ = Fc_palmitate_R_palmitate_+Fc_lactate_R_lactate_+Fc_pyruvate_R_pyruvate_+ Fc_glucose_R_glucose_+*y*Ra, where the Fcs are fractional contributions for each substrate determined by isotopomer analysis and R is an assumed respiratory quotient (R_palmitate_ = 2.8, R_lactate_ = 3, R_pyruvte_ = 3, and R_glucose_ = 2.9). *y*Ra represents the anaplerotic component. The calculated citric acid cycle_flux_ was normalized for each substrate by dividing the total citric acid cycle_flux_ by the number of acetyl-CoA esters yielded per molecule of that substrate (palmitate = 8, lactate = 1, glucose = 2, pyruvate = 1) and multiplying with the corresponding Fc. The unlabeled Fc was used for the palmitate Fc in this formula for reasons noted in the Discussion.

### Immunoblotting

Fifty micrograms of total protein extract from mouse heart tissue was electrophoresed along with a lane of molecular weight size markers (Novex Sharp Pre-stained, Invitrogen) in 4.5% stacking and 10% resolving SDS-polyacrylamide gels. The gels were then electroblotted onto PVDF-Plus membranes. The Western blot was blocked for 1 h at room temperature with 5% nonfat milk in Tris-buffered saline (TBS) plus Tween 20 (TBST; 10 mM Tris·HCl, pH 7.5, 150 mM NaCl, and 0.05% Tween 20), followed by an incubation with primary antibody diluted in the above blocking solution. After four 5-min washes with TBST, the membrane was incubated at room temperature for 1 h with the appropriate IgG secondary antibody conjugated to horseradish peroxidase (HRP). The membranes were rinsed four times for 5 min with TBST and visualized with enhanced chemiluminescence upon exposure to Kodak.

BioMax Light ML-1 film. Membranes were stripped by washing for 30 min with 100 mM 2-mercaptoethanol, 2% (wt/vol) SDS, 62.5 mM Tris·HCl, pH 6.7, at 70°C, followed by three 10-min washes with TBS for additional antibody analysis. All immunoblots were normalized to α-tubulin protein (Santa Cruz Biotechnology, Inc, Santa Cruz, CA) levels, which are shown. The primary antibodies used in this study were PPARα (Santa Cruz Biotechnology, Inc, Santa Cruz, CA),PDK4 (personal gift from Robert Harris, Indiana University School of Medicine, Indianapolis, IN), CD36 (Novus Biologicals, Littleton, CO), liver (l)- and muscle (m) specific carnitine palmitoyltransferase 1 (lCPT-1 and mCPT-1; personal gifts from Gebre Woldegiorgis, Oregon Health Sciences University, Beaverton, OR).

### RNA Extraction, Labeling, and Quality Control

Total mRNA was extracted from the frozen heart tissue using Trizol (Life Technologies, NY). Contaminating DNA was removed by deoxyribonuclease I (Thermo Scientific, MA) digestion of the RNA. An average of 15–20 µg of total RNA was obtained from 20 mg of frozen mouse heart tissue. Quantity, OD260/280 and OD 260/230, of total RNA was assessed by UV spectrophotometer.

### RT-qPCR Analyses

Primers targeting CD36 (PPM03796D-200), Actin (PPM02945B-200), 18S (72041A-200), muscle specific CPT-1(PPM57688A-200), liver specific CPT-1 (PPM25930C-200), long-chain acyl coenzyme A synthetase (ACSL1; PPM33300G-200) were purchased from Qiagen, CA. First strand cDNA synthesis was performed using the iScript™ cDNA synthesis kit (Bio-Rad, CA). The second step quantitative PCR was performed using RT^2^ SYBR Green Fluor qPCR Mastermix (Qiagen, CA).

The level of transcripts for the constitutive housekeeping gene products 18S rRNA and actin was quantitatively measured in each sample to control for sample-to-sample differences in RNA concentration. PCR data are reported as fold difference from Old using the gene expression analysis methodology [21]. The rt-qPCR was performed using the Bio-Rad icycler iQ.

### Statistical Analysis

Reported values are means ± standard error (SE) in figures and text. Data were analyzed with a single factor ANOVA for multiple comparisons. If significance was identified between the groups by ANOVA (P-value <0.05); then a two-way unpaired t-test was performed. A two way unpaired t-test was performed for Western blots and RT-qPCR. Paired t-tests were used to compare serial echocardiographic measurements, TH levels and free fatty acids within individual mice. Criterion for significance was p<0.05 for all comparisons.

## Results

### Morphometric and Echocardiographic Data

Prolonged exposure to high TH levels can cause pathologic hypertrophy [22], [23]. We assessed for this with morphometric and echocardiographic studies. TH supplementation did not change heart weight to body weight ratios in Old mice (Old 4.9±0.5 mg/g versus Old-Th 4.8±0.3 mg/g, P>0.05). There were no changes in the interventricular septum or posterior wall thickness compared to baseline (pre-treatment) with TH supplementation (Table 1). Accordingly, our TH dosing did not cause hypertrophy. Echocardiographic measures of systolic and diastolic function were also unchanged from baseline (Table 1). Of note, these were sedated echocardiograms in the unstressed state. The heart rate measured during the echocardiograms trended toward being increased after TH supplementation (p = 0.085).

**Table 1:** Echocardiographic analysis of the left ventricle at baseline and after thyroid hormone treatment.

	Baseline	TH
HR, BPM	453±19	510±16
MV A wave, mm/s	421±59	471±33
MV E Wave, mm/s	574±74	626±24
MV E/A ratio	1.4±0.2	1.3±0.1
IVST, mm	0.86±0.05	0.87±0.01
EDD, mm	3.9±0.1	4.0±0.0
ESD, mm	2.7±0.1	2.8±0.1
PWT, mm	1.0±0.1	1.1±0.1
LV%FS	30±2	30±1

HR, heart rate; BPM, beats per minute; MV, mitral valve; IVST, interventricular septal thickness; EDD, end diastolic dimension; ESD, end systolic dimension; PWT, posterior wall thickness; LV%FS, left ventricular per cent fractional shortening. Values are means±SEM. n = 4 paired mice.

### Circulating Thyroids Hormone and Free Fatty Acid Levels

We determined plasma thyroid hormone levels before and after 3 weeks of Thyroid Powder administration (n = 4–5 mice). T3 is that active form of thyroid hormone. TH supplementation significantly increased paired plasma T3 from 0.8±0.1 ng/ml at baseline to 1.2±0.2 ng/ml after treatment (p<0.05). T4 is the precursor to T3 [24]. T4 levels significantly decreased from 35.0±0.5 ng/ml before treatment to 15.0±1.7 ng/ml after supplementation (p<0.05) suggesting inhibition of the pituitary-thyroid axis by our exogenous thyroid hormone supplementation. Circulating free fatty acid levels did not change from baseline (baseline 997±280 µM versus TH supplementation 1073±322 µM).

### Cardiac Function during the Working Heart Experiments

*Ex Vivo* functional assessments were made during the working heart perfusions. All reported values are after 20 minutes of stable left atrial infusion. At high afterload, -dP/dT was decreased in Old versus Young (Table 2). TH not only abrogated this decrease in –dP/dT but significantly increased +dP/dT _max_ compared to Young and improved multiple measures of cardiac function in comparison to the Old group (Table 2). Of note, TH did not increase heart rate in the isolated heart perfusions. For comparison, we performed a limited number of working heart experiments at standard afterload in the Young and Old mice only. There were no functional differences between the groups at standard afterload with the exception of decreased myocardial oxygen consumption in Old (Table 3).

**Table 2:** Functional measurement during isolated working heart with ^13^C-labeled substrates at high afterload (80 mmHg).

	Young	Old	Old-TH
Heart rate (BPM)	414±16	432±13	407±18
+dP/dT_max_ (mmHg/sec)	5582±177*	5154±190*	6531±255
-dP/dT_min_ (mmHg/sec)	−5174±123	−4542±198* #	−5507±201
Power (ml/min)*mmHg	1483±151	1260±131*	1701±130
Aortic flow (ml/min)	9.3±0.3	8.0±0.7	10.7±0.6
Coronary flow (ml/min)	5.3±1.0	4.5±0.5	5.4±0.6
MVO_2_ (µmol/g wet weight/min)	11.7±1.0	9.8±1.0	11.2±0.8
Efficiency (ml*mmHg/µmol O_2_/g)	121±10*	130±8*	154±8

Values are means±SEM. N = 7–9 per group.

* p<0.05 versus Old-TH,

# p<0.05 versus Young.

**Table 3:** Functional measurement during isolated working heart with ^13^C-labeled substrates at standard afterload (50 mmHg).

	Young	Old
Heart rate(BPM)	404±11	357±21
+dP/dT_max_ (mmHg/sec)	4060±195	4426±423
-dP/dT_min_ (mmHg/sec)	−3684±124	−4034±243
Power (ml/min)*mmHg	1280±176	1273±102
Aortic flow (ml/min)	10.8±1.1	10.0±0.1
Coronary flow (ml/min)	3.9±0.7	3.5±0.3
MVO_2_ (µmol/g wet weight/min)	9.8±0.5	6.8±0.2*
Efficiency (ml*mmHg/µmol O_2_/g)	149±6	188±15

Efficiency is defined as cardiac power/oxygen consumption. MVO2, Myocardial oxygen consumption. Values are means±SEM. N = 4 per group.

* p<0.05.

### Fc of Acetyl-CoA to the Citric Acid Cycle and Substrate Flux

**Figure 1:**
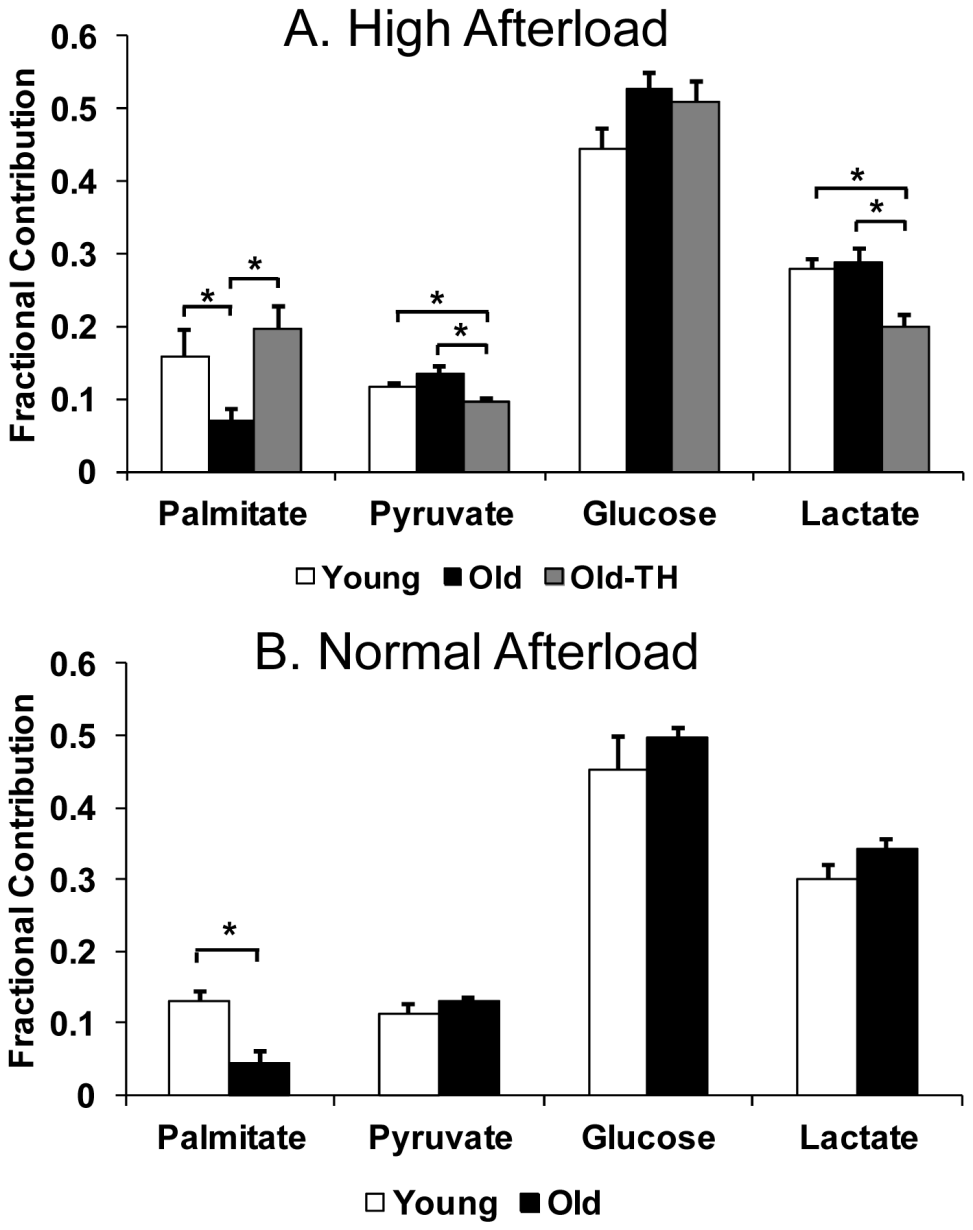
Fractional contribution (Fc) to the citric acid cycle for unlabeled substrates (predominately palmitate), pyruvate, glucose and lactate. (A) Young, Old and Old-TH at high afterload (n = 7–9 per group). (B) Young and Old mice at standard afterload (n = 4–5 per group). Values are means±SEM. *p<0.05 between indicated groups.

We determined the Fc of acetyl-CoA to the citric acid cycle for each studied substrate. Of note, glycolytic breakdown of [1-^13^C] glucose generates one labeled and one unlabeled acetyl-CoA molecule. The reported glucose Fc mathematically accounts for this fact. Since the remaining unlabeled Fc is predominately palmitate, we reported this value as palmitate Fc. As noted, we wanted to determine whether acutely increasing afterload affects substrate contribution to the citric acid cycle (Figure 1B). Palmitate Fc was decreased in Old versus Young with no significant changes in the other substrates. Thyroid hormone supplementation (Old-TH) increased palmitate Fc compared to Old alone. Pyruvate and lactate Fc were both significantly decreased in Old-TH versus Young and Old. Overall citric acid cycle flux was greatest in the Young (Table 4). There was a trend towards increased CAC flux in Old-TH versus Old (p = 0.1). Thyroid hormone increased palmitate flux to similar levels as the Young group. However, lactate flux was reduced in both Old and Old-TH groups compared to Young and also in Old-TH versus Old.

**Table 4:** Flux rates for total citric acid cycle (CAC) and individual substrates at high afterload.

	Young	Old	Old-TH
CAC	4.3±0.1	3.1±0.2*	3.7±0.2*
Palmitate	0.09±0.03	0.03±0.01* #	0.09±0.00
Pyruvate	0.48±0.03	0.43±0.03	0.34±0.02* $
Glucose	0.9±0.1	0.8±0.1	0.9±0.1
Lactate	1.2±0.1	0.9±0.0*	0.8±0.1*

Units are µmol/g/min. Values are means±SEM. n = 5–8 per group.

* p<0.05 versus Young;

# p<0.05 versus Old-TH;

$ p<0.05 versus Old.

We also performed metabolic studies at the standard afterload in Young and Old mice (Figure 1B). Palmitate Fc was significantly decreased in Old versus Young. Although the individual carbohydrates did not differ between groups, the total carbohydrate Fc (calculated as lactate Fc+glucose Fc+pyruvate Fc) was significantly greater in Old compared to Young (0.94±0.01 versus 0.87±0.02, respectively). Overall citric acid cycle flux was decreased in the Old mice which led to diminished palmitate and glucose flux (Table 5).

**Table 5:** Flux rates for total citric acid cycle (CAC) and individual substrates at standard afterload.

	Young	Old
CAC	3.4±0.2*	2.4±0.1
Palmitate	0.06±0.01*	0.02±0.00
Pyruvate	0.37±0.01	0.32±0.06
Glucose	0.8±0.1*	0.5±0.0
Lactate	1.0±0.0	0.8±0.1

Units are µmol/g/min. Values are means±SEM. n = 4 per group. * p<0.05.

### Protein Expression and RT-qPCR

**Figure 2:**
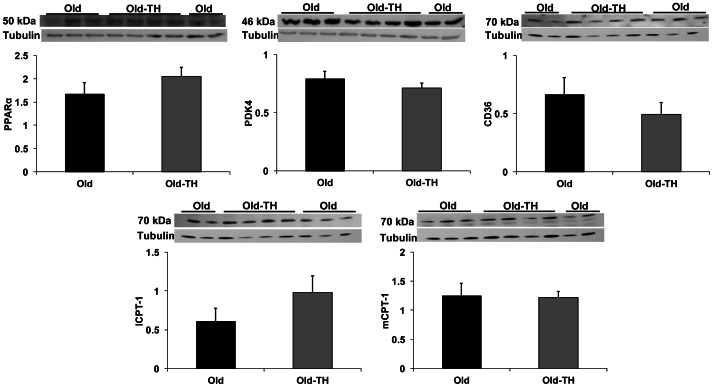
Expression of proteins involved in the regulation of fatty acid metabolism . All values are arbitrary units normalized to α-tubulin (tubulin) as shown in the figure. The approximate molecular weight of each protein is noted. n = 4–5 per group. No differences were found between the groups for any protein. PPAR, peroxisome proliferator-activated receptor; PDK, pyruvate dehydrogenase kinase; lCPT-I, liver-specific carnitine palmitoyltransferase 1; mCPT-1, muscle-specific CPT-1. Note that CD36 and lCPT-1 were run on the same membrane after antibody stripping.

**Figure 3:**
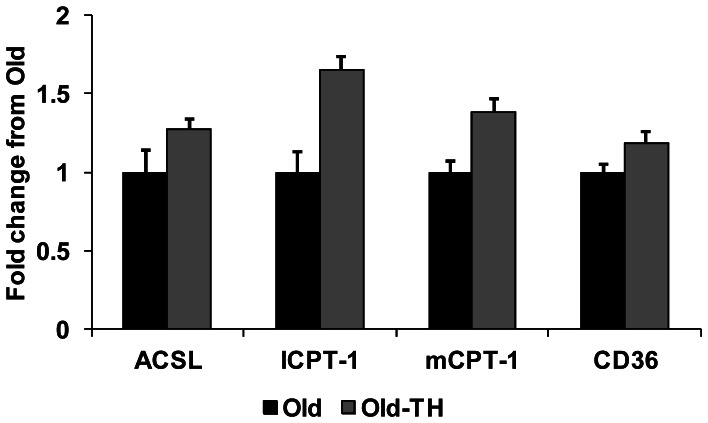
Determination of steady-state mRNA levels for genes involved in fatty acid metabolism. All values are fold difference from Old. N = 4 per group. No differences were found between the groups. ACLS, long-chain acyl coenzyme A synthetase; lCPT-1, liver-specific carnitine palmitoyltransferase 1; mCPT-1, muscle-specific CPT-1.

Our prior study showed that the metabolic changes from aging were associated with decreased total protein levels of PPARα and PDK4 [14]. Accordingly, we determined whether the metabolic changes from TH were related to changes in these proteins. As shown in Figure 2, TH did not alter total protein levels of either. The fatty acid cellular membrane transport protein CD36 and mitochondria transporter CPT-1 (both mCPT-1 and lCPT-1) protein levels were also unchanged. Further, RT-PCR did not detect any differences in steady-state mRNA expression of CD36, mCPT-1 and lCPT-1 as well as long-chain acyl coenzyme A synthetase (Figure 3).

## Discussion

Our primary objective was to test the hypothesis that disturbances in thyroid hormone homeostasis mediate the metabolic phenotype in aging hearts and contribute to cardiac dysfunction. Specifically, we investigated whether TH treatment alters oxidative metabolism in aged mice and whether this could improve the myocardial response to increased stress. We reconfirmed that aging impairs fatty acid utilization and oxidation at standard afterload. The aged heart maintained cardiac function during the ex vivo working heart evaluations. Accordingly, we focused on stressing the aged heart to investigate the metabolic and functional changes, which might occur at higher workload. Thus, we acutely increased afterload during working heart perfusions from 50 mmHg to 80 mmHg. Functionally, aging reduced –dP/dT compared to Young, while other recorded functional measurements were similar. The aged heart exhibited reduced diastolic function that is apparent only when stressed. This result is in accordance with the finding that diastolic relaxation abnormalities are typically the earliest manifestation of cardiac dysfunction in aged human hearts [25]. These changes in diastolic function have been attributed to down-regulation of phospholamban and upregulation of SERCA [26]. Koonen et al. also acutely increased afterload from 50 mmHg to 70 mmHg in aged hearts and found that both systolic and diastolic function was reduced in aged mice compared to controls [12]. However, they included isoproterenol in the perfusate which may account for the differences from the current study.

We previously showed that fatty acid oxidation is impaired in aged mice during standard afterload conditions [14]. Elucidation of carbohydrate contributions to the citric acid cycle was a focus of the current study. Therefore, we did not specifically label palmitate, as we can only discriminate three labels using the isotopic analyses. However, the unlabeled Fc for Young and Old were both similar to the free fatty acid Fc in our prior study when we perfused with glucose and ^13^C labeled free fatty acids, ketones, and lactate [14]. Thus, we logically assumed that this unlabeled fraction represented the palmitate contribution. The reduction in this unlabeled (palmitate) Fc persisted during the acute elevations in afterload. Due to the ^13^C labeling considerations discussed earlier, ketones were not supplied in the current experiments. To make up for the lack of ketones, Young and Old increased carbohydrate utilization. Overall citric acid cycle flux decreased with aging primarily due to reduced palmitate flux. The reduced citric acid cycle flux could decrease ATP production and contribute to the mildly abnormal diastolic function with aging. The totality of this data suggests that metabolic changes from aging may mildly affect cardiac function.

Our previous studies suggested that mild thyroid hormone deficiency in aged mice inhibits fatty acid oxidation through transcriptionally mediated downregulation of PPARα [14]. The alterations in PPARα in turn suppress PDK4, which should then disinhibit pyruvate dehydrogenase complex by lack of phosphorylating enzyme. However, the prior study used L-lactic-3-[^13^C] acid as the primary carbohydrate tracer. In the current experiments, we sought to determine if other specific differences in the carbohydrate pathways occur and are responsible for changes in carbohydrate flux in aging, or if these modifications are ameliorated by thyroid hormone supplementation. Thus, we used a complex labeling pattern which reveals individual fractional contributions from glucose, pyruvate, and lactate. The data indicate that the aging heart uniformly maintains flux through these carbohydrates during transition to high work. The inability to increase total citric acid cycle flux rests with continued marked suppression of fatty acid oxidation.

The primary focus of our current study was whether TH supplementation reverses aging-induced changes in myocardial metabolism and function. This is clinically relevant for the problem of subclinical hypothyroidism and heart failure. Our previous work demonstrated a nearly 30% reduction in total T3 levels in aged mice [14]. T3 is the active form of thyroid hormone. Our supplementation protocol successfully increased plasma T3 levels. It is unlikely that we caused important hyperthyroidism since the echocardiographic and morphometric data did not demonstrate cardiac hypertrophy. Thyroid hormone supplementation also suppressed T4 levels. Chen et al. also showed that pharmacologically elevating T3 levels suppresses T4 in rats presumably by inhibiting the pituitary-thyroid axis [24].

TH did not alter cardiac function in vivo as measured by sedated (unstressed) echocardiograms. This was not surprising since we did not detect functional differences between the Young and Old groups ex vivo at standard afterload. However, our ex vivo analysis at high afterload showed that multiple measures of cardiac function improved with TH. The Old-TH group even had greater dP/dT and efficiency of oxygen utilization compared to Young. TH supplementation clearly reversed the aging-induced cardiac dysfunction apparent at high afterload. Cardiac oxidative metabolism was also altered by TH supplementation. Old-TH palmitate Fc and flux were significantly increased to a value similar to the Young group. Thus, the improvement in cardiac function was accompanied by increased fatty acid oxidation and a trend toward increased citric acid cycle flux. Thyroid hormone also appears to selectively suppress pyruvate and lactate oxidation. These metabolic changes with TH supplementation are consistent with previous findings from our lab where rat thyroidectomy selectively decreased free fatty acid flux. T3 rapidly altered substrate flux and fractional contributions by shifting utilization patterns away from the pyruvate dehydrogenase pathway and enhancing fatty acid oxidation in both hypothyroid and euthyroid rats [27], [28]. Further, acute thyroid hormone administration reduced lactate Fc and flux in these studies as well. Pyruvate was not previously evaluated.

TH supplementation could improve cardiac function through multiple mechanisms including changes in the excitation-contraction and transport proteins discussed earlier. Augmenting fatty acid oxidation may also be beneficial. For example, Kolwicz et al. recently showed that preventing the decline in fatty acid oxidation during pressure overload hypertrophy preserves cardiac function [29]. Work from our laboratory showed that fatty acid oxidation is increased in c-Myc induced compensated hypertrophy [16]. The mechanisms responsible for this favorable response are incompletely understood. Fatty acids are more carbon efficient and produce more ATP per mole than carbohydrates. Therefore increased carbohydrate reliance could result in an energy deficient state and impaired function [30]. Accordingly, TH supplementation may have improved myocardial energy status and made the hearts more tolerant to stress. Future studies are indicated to determine whether mild TH supplementation can also increase fatty acid oxidation in non-aged hearts subjected to stressors such as aortic constriction or myocardial infarction.

### Limitations

We were unable to identify the mechanisms for the changes in cardiac metabolism with TH. Our previous work showed that aging reduced protein levels of PPARα and PDK4; which would be consistent with decreased fatty acid oxidation. However, levels of these proteins were not altered by TH. Fatty acid oxidation is regulated at multiple steps from cellular uptake to mitochondrial transport to β-oxidation [31]. Kooken et al found that fatty acid uptake is greater in aged mice that younger mice even though fatty acid oxidation is limited [12]. Therefore, it is doubtful that TH mediates its metabolic effects through further increases in fatty acid uptake. In support of this, we did not find changes in CD36 protein and steady-state mRNA levels. There were also no differences in steady state mRNA levels of lCPT-1, mCPT-1 and ACLS as well as protein levels of mCPT-1 and lCPT-1. TH supplementation also decreased lactate and pyruvate utilization without affecting glucose oxidation. This suggests that the TH-mediated effect on carbohydrate oxidation does not occur at the pyruvate dehydrogenase complex, since this should affect all carbohydrates to a similar degree. Additional work is necessary to determine whether this metabolic phenotype is due to post-translational modifications or if other enzymes are transcriptionally affected by TH.

### Conclusions

Aging causes a decrease in fatty acid oxidation which is associated with mildly reduced cardiac function at high afterload. The aged heart uniformly increases utilization of lactate, glucose and pyruvate in place of the fatty acids. TH supplementation increases fatty acid oxidation while improving measures of cardiac function. These results suggest an important metabolic effect from TH supplementation in aged mice, which improves functional capacity at high workload.
